# The Consequences of Habitual Rumination, Expressive Suppression, and Perceived Stress on Mental and Physical Health Among Older Adults

**DOI:** 10.3390/geriatrics10050114

**Published:** 2025-08-25

**Authors:** Eyal Gringart, Rodrigo Becerra, Andrea Smith

**Affiliations:** 1 School of Arts and Humanities, Edith Cowan University, 270 Joondalup Drive, Joondalup 6027, Australia; 2 School of Psychological Science, The University of Western Australia, 35 Stirling Highway, Perth 6009, Australia; 3 Conscious Psychology, 142 Giles Avenue, Padbury 6025, Australia; admin@coscious-psychology.com.au

**Keywords:** rumination, suppression, perceived stress, health, well-being, depression, anxiety

## Abstract

**Background/Objectives:** The current study aimed to investigate whether habitual rumination, suppression, and perceived stress predict poor mental and physical health as well as well-being in a group of older adults (aged 50 to 80 years) from a non-clinical community sample. **Methods:** The current study comprised a cross-sectional survey design with online self-report measures. It was predicted that higher levels of rumination, suppression, and perceived stress would predict lower levels of general health as well as well-being, and heightened levels of depression and anxiety. **Results:** Findings from the study indicated that both rumination and perceived stress significantly predicted heightened anxiety, heightened depression, and decreased physical health as well as well-being. **Conclusions:** These results replicate and extend past research on rumination. However, diverging from past research, suppression was not a significant predictor, or correlate, of stress, anxiety, or of general health and well-being; though, suppression did weakly but significantly predict depression.

## 1. The Consequences of Habitual Rumination and Expressive Suppression, as Well as Perceived Stress on Mental and Physical Health Among Older Adults

For many years, there has been a focus on studying various emotion regulation styles and their potential influence on mental and physical health as well as well-being, and in some cases, their potential involvement in the aetiology of mental and physical illness. Suppression and rumination are considered to be generally maladaptive styles of emotion regulation. More specifically, some researchers, e.g., [1], have highlighted the negative impacts of the suppression of emotional expression on both mental and physical health whilst others, e.g., [2,3], have focused on the negative consequences of rumination on mental and physical health. Additionally, the chronic use of rumination and suppression have been suggested to have long term cumulative effects on mental and physical health, putatively, due to the heightened and prolonged stress-responses [1,3,4,5,6,7,8,9,10,11].

Past research, e.g., [1], has primarily focused on experimental designs with manipulations or inductions of rumination and suppression and hence emphasised the short-term effects of rumination and suppression rather than their long-term effects. Additionally, many of the studies that suggested associations between rumination, suppression, and increased psychopathology, focused specifically on college and university student-age populations, or clinical-only samples, which limit the generalisability of their findings, e.g., [3,11].

The current study proposed that assessing this potential relationship in older populations and community samples could be of particular value. This is because the effects of rumination, suppression and stress may have long term cumulative effects, and as such, ailments influenced by rumination, suppression and stress are more likely to become apparent among samples of older adults compared to the typical university-aged samples typically used in such research. The current study, therefore, aimed to investigate whether habitually engaging rumination and suppression, and high levels of perceived stress predict poor mental and physical health as well as well-being.

## 2. Literature Review

### 2.1. Suppresion

Expressive suppression, which involves the attempt to hide, inhibit or reduce emotion-expressive behaviour is generally considered to be a maladaptive emotion regulation strategy [6,12]. Such suppression may be accomplished by inhibiting facial, vocal, and behavioural cues, as well as avoiding the verbal expression of emotional experience to others. For the purposes of the current paper, and in keeping with Gross’s definition of suppression as measured by the Emotion Regulation Questionnaire (ERQ), which was used by the current study, expressive suppression is referred to simply as ‘suppression’.

Ref. [3] found that people who frequently used suppression were more likely to experience negative emotions, that suppression was significantly positively correlated with self-reported symptoms of depression, and negatively correlated with life satisfaction, well-being, optimism, and self-esteem. Additional studies similarly found that regular use of suppression related to poor interpersonal behaviour and less social and emotional support, as well as lower levels of well-being and life satisfaction [13,14,15,16,17]. Further, Individuals high on trait suppression also experience intensified and prolonged physiological responding to stress and increased sympathetic activation of the cardiovascular system following inhibition of emotional expression [1,6,7].

### 2.2. Rumination

Ref. [2] defined rumination as a response pattern to distress wherein the individual repetitively and uncontrollably focuses on negative and distressing content. This occurs whilst failing to take active steps towards resolving or altering the source of the distress.

Rumination has been associated with a variety of negative or maladaptive physical and physiological outcomes. [18] surveyed older adults with an average age of 69 years and found that ruminators reported poorer physical health, as well as a greater predisposition to heightened levels of arousal.

Rumination has also been associated with increased physiological responses to stressors, including delayed cortisol recovery and delayed cardiovascular recovery after exposure to stress [5,8,10,11]. The importance of the association between rumination and poor physiological recovery is heightened by the findings of [9] that ruminators experienced greater increases in the number of stressful events over a period of one year when compared to non-ruminators.

Both rumination and suppression have been found to have significant impact on physiological stress responses in the forms of intensified and prolonged physiological responding to stress (suppression and rumination), increased sympathetic activation of the cardiovascular system following inhibition of emotional expression (suppression), and delayed cortisol and cardiovascular recovery after exposure to stress (rumination). More specifically, studies have found that rumination can generate and extend the stress response [19,20]. However, the results from these and other similar studies generally assessed the relationship between physiological markers of stress and induced rumination and suppression, rather than trait rumination and suppression.

In respect to trait rumination, some studies have found that higher levels of perceived stress and stressful experiences predict increases in rumination [18,21,22,23,24] and equally, that rumination can both generate and extend the stress response [19,20,23].

Similarly, the emotion regulation style of suppression has also been found to be significantly correlated with increases in stress, however this has primarily been measured with physiological markers for stress, rather than subjective self-ratings of perceived stress. Interestingly, a 2008 study [25] found that trait suppression was indeed associated with higher self-reported stress symptoms.

Researchers have suggested that one of the reasons suppression is associated with higher levels of stress and psychopathology may be an effect of the costly nature of engaging in suppression, particularly in respect to the constant self-monitoring and self-control required to limit the expression of genuine emotion. In this sense, the association between suppression, stress, psychopathology and/or increased risk of poor health outcomes may be explained using a diathesis-stress model. That is, a tendency to suppress the expression of emotions could be considered a diathesis, which is associated with increased and prolonged stress responding (stressor) and leads to increased risk of poor mental and physical health.

The same model could be considered in respect to the association between rumination, stress, and negative mental and physical health outcomes. Taken together, the association between the tendency to ruminate (diathesis) and experience heightened levels of stress may increase the likelihood of psychopathology and risk of poor health. This model was used as the theoretical backdrop for the current study and is in line with [21] who explored the association between rumination, perceived stress, and posttraumatic stress disorder.

As can be seen from the above review, research suggested a significant association between rumination/suppression and reduced mental health and well-being, specifically in the form of depression, general anxiety, social anxiety, low levels of emotional well-being, reduced positive affect and increased negative affect, as well as increases in eating disorders and substance abuse. Previous research has also suggested a significant association between stress and rumination/suppression.

Although investigators have suggested associations between emotion regulation styles and broad health outcomes, research has primarily focused on cardiovascular health risk factors [4] and other physiological markers for stress. Considering that suppression is significantly correlated with increases in the experience of negative affect along with findings that negative emotion is associated with decreased lung function [26], onset of cancer [27] and several other diseases, suggest an association between suppression and the development of cardiovascular disease [28]. However, prominent researchers in the field of suppression have explicitly referred to the significant gap in the literature when considering the association between suppression and more general health outcomes, which have not yet been researched comprehensively [29].

Similarly, research on the association between rumination and physical health has primarily been conducted experimentally, the findings of which have suggested a potential association between rumination and physical health via delayed post-stress recovery and an increased risk of developing hypertension [3,30]. There is a considerable gap in the literature when it comes to assessing the association between trait rumination and physical health outcomes in non-experimental settings.

Considering this gap in the literature in respect to the associations between both rumination and suppression with general health outcomes, the current study aimed to contribute to the existing body of knowledge by including measures of general health and well-being, along with mental health measures.

### 2.3. The Current Study

The current study aimed to investigate whether habitually engaging in rumination and suppression predicts poorer mental and physical health as well as well-being as a potential long-term consequence of these maladaptive emotion regulation styles. With the Diathesis-Stress Model in mind, we also wanted to investigate the role of perceived stress, as both rumination and suppression have been associated with psychological and emotional stress responses, and stress is also associated with poor mental and physical health outcomes [31,32,33]. Additionally, whilst both rumination and suppression have been associated with increased self-reported perceived stress levels, there are few studies that have explored this association explicitly. This gap in the literature informed our decision to include a measure of perceived stress to investigate whether these associations could be replicated, i.e., whether there were indeed significant correlations between rumination and suppression with perceived stress. Furthermore, by including a measure of perceived stress, we were able to assess whether each of these variables (rumination/suppression and perceived stress) would significantly predict mental and physical health outcomes, potentially lending support to the Diathesis-Stress Model.

Well-validated self-report measures were used for trait rumination, trait suppression and perceived stress, in addition to self-report measures of anxiety, depression, and general health and well-being. The study focused on trait rumination, suppression, and perceived stress, and their potential cumulative effects over time on mental and physical health and well-being. As we hypothesised that the effects of rumination, suppression and stress have long-term cumulative effects, we recruited participants who were in middle to late adulthood, as we were interested primarily in the effects of habitual rumination and/or suppression over time.

For both mental and physical health, participants were asked to indicate whether they had an official current diagnosis, a previous but not current diagnosis, or had never had a mental/physical health diagnosis. This information was used to group participants into three groups according to their diagnostic status for both physical and mental health, in order to then see whether this had a significant effect on any of the variables of interest, particularly that of rumination, suppression, and perceived stress.

Four hypotheses were posed:

Hypothesis One: It was hypothesized that low scores on general health and well-being (lower scores indicating poorer health) would be correlated with high scores on trait rumination, suppression, and perceived stress.

Secondary hypotheses

Hypothesis Two: It was predicted that high scores on depression and anxiety would each be positively correlated with high scores on trait rumination, suppression, and perceived stress.

Hypothesis Three: It was hypothesized that lower scores on general health and well-being (lower scores indicating poorer health) would be significantly predicted by higher scores on trait rumination, suppression, and perceived stress.

Hypothesis Four: It was hypothesized that depression and anxiety would be predicted by higher trait rumination, suppression, and perceived stress.

## 3. Method

### 3.1. Research Design

The current study employed an online quantitative cross-sectional survey design. The study was conducted using a selection of self-report inventories, which were accessed by participants through an online webpage link. Relationships between the following variables were investigated: rumination, perceived stress, suppression, anxiety, depression, and general health and well-being. Using the formula *n* = 50 + 8(k), the minimum number of participants appropriate for standard multiple regression analysis with 5 predictors is 90. We, thus, aimed to recruit a minimum of 90 participants.

### 3.2. Participants

A total of 121 out of 125 participants fully completed the online survey (78 females; 43 males). The Inclusion criterion for participation was that participants would be between the ages of 50 and 80 years. The participants ranged in age from 50 to 76 with a mean age of 59 (SD = 6.54). In terms of marital status, 72 participants identified as married, 25 divorced, 11 single (never married), 8 widowed, 3 separated, and 2 in a de-facto/common-law relationship. Parameters were set regarding the geographical location of survey respondents to limit the study to participants from a primarily western, individualistic culture, therefore participants were from the following countries: Australia, Canada, Great Britain, and the Unites States. Unfortunately, we are unable to report on the specific geographical distribution of respondents as this information was not collected by the survey.

Participants were recruited anonymously through the Mechanical Turk platform and were paid a nominal fee ($0.55 AUS) for participation in the 15-min survey, based on the standard pay scale for Mechanical Turk. The participants who were not recruited through Mechanical Turk consisted of anonymous volunteers that were recruited through snowball sampling using the researcher’s personal social media (Facebook) and through an advertisement in the Council of the Ageing (COTA) e-newsletter.

Participants were asked whether they had any current or past mental health diagnoses, of which 75.2% (91) reported to have never had a mental health diagnosis, 15.7% (19) having had a previous, but not current diagnosis, and 9.1% (11) reported to have a current mental health diagnosis. A breakdown of the mental health diagnoses for those with current or previous diagnoses is presented in Table 1.

Participants were also asked about physical health diagnoses, with 49.6% (60) reporting a history of no diagnoses, 40.5% (49) reporting a current physical diagnosis, and 9.9% (12) reporting a previous but not current diagnosis. Specific types of physical health diagnoses and their frequencies amongst participants are presented in Table 1.

## 4. Materials

The scales and demographic questions were combined to form an online survey using Qualtrics. The materials comprised a demographic questionnaire, which included nine questions relating to general demographics, as well as mental and physical health diagnoses. A compilation of validated scales was used to measure the variables of interest, namely rumination, suppression, general health and well-being, depression, anxiety and perceived stress.

General health and well-being were assessed using the Short Form 36 Health Survey (SF36). Answers to each question are scored and then summed to produce raw scale scores for each health-related construct, which are then transformed to a 0–100 scale. There are 36 items, each of which either contribute to the Physical Component Summary or the Mental Component Summary. A sample item from the survey is: “In general, would you say your health is: 1—excellent; 2—very good; 3—good; 4—fair; 5—poor”. The SF36 showed satisfactory internal consistency with α = 0.79 [34].

Expressive Suppression was measured using the Emotion Regulation Questionnaire (ERQ). The ERQ was designed to assess individual differences in the habitual use of two emotion regulation strategies: suppression and cognitive reappraisal. The current study only used the suppression subscale, which consisted of 4 items scored on a 7-point Likert-type scale ranging from 1 (strongly disagree) to 7 (strongly agree). A sample item from the questionnaire is: “I control my emotions by not expressing them”. The ERQ showed satisfactory internal consistency with α = 0.73 [12].

Rumination was measured with the Ruminative Responses Scale (RRS), which is a 10-item short form self-report scale assessing how often people ruminate based on a 4-point Likert scale from 1 (almost never) to 4 (almost always). A sample item from this scale is “think about how you don’t feel up to doing anything”. The RRS showed satisfactory internal consistency with α = 0.76 [35].

Perceived Stress was assessed with the Perceived Stress Scale (PSS). It is a 10 item self-report measure of perceived stress within the past month and is scored using a 4-point Likert scale. A sample item from this scale is “in the last month, how often have you been able to control irritations in your life?” The PSS showed strong internal consistency ranging between α = 0.84–0.86 [36].

Depression and anxiety were assessed using part of the Depression, Anxiety and Stress Scales 21 (DASS 21). The 14 items assessing depression and anxiety were used and the stress items were excluded as stress was measured elsewhere (in the PSS). The DASS is based on a 4-point Likert scale from 0 (did not apply to me at all) to 3 (applied to me very much, or most of the time). A sample item from this scale is “I felt that life was meaningless”. The DASS 21 showed satisfactory internal consistency with α = 0.75 depression; and α = 0.74 anxiety [33].

In total, there were 86 items across the six measures. These scales were selected as the literature reported that they have good reliability and validity and because they are widely used.

### 4.1. Procedure

Prior to commencing research, approval from the University’s Research Ethics Committee was obtained (17492 SAUNDERS). The survey was completed anonymously online through a webpage link. Before the survey commenced, respondents viewed an electronic informed consent form, which contained information about the purpose of the project, the procedure of participation, and the benefits and risks of participating. Participants were asked to indicate that they had read and understood the information provided, and agreed to participate in the study as described, before they proceeded to the survey. They were also provided with contact details for support services if they experienced distress because of their participation, and were advised that they could discontinue the study at any time. Participation in the survey took an average of 16 min. After the participants completed all items, a final page was displayed, which thanked them for their participation and explained the implications of the research being done. This final page also provided participants with support resources to contact if they felt that the questionnaire had raised any unsettling emotions for which they required support.

### 4.2. Analysis

Data were coded as well as screened for normality and for multivariate outliers. Several statistical analyses were run, including correlations, multiple regressions, MANOVAS, and independent samples *t*-tests.

## 5. Results

Data were inputted into SPSS (version 24) from Qualtrics, coded and screened for missing data, which led to the deletion of three incomplete data sets. Data were then screened for multivariate outliers using Mahalanobis distance for 13 variables (including subscales of measures) with critical chi square value of 34.53 at 0.001 level of significance, which led to the identification and deletion of three multivariate outliers. Two rounds of screening for univariate outliers, which were adjusted by substituting their values with the outermost corresponding value in their direction that was allowed by the 95% confidence interval around their mean. 

The distributions of depression, anxiety, and, to a lesser extent, perceived stress, were negatively skewed. Contrastingly, general health and well-being was positively skewed. As the sample size facilitated robust statistical analyses, however, these posed minimal threat in the current study.

### Analyses

Average scale scores. Across the 121 participants, the mean score on the general health and well-being scale (SF36) was 72.95 (SD = 20.51), out of a possible range of 0–100 with 100 indicating self-reported “excellent” health. The mean score on the DASS was 3.05 (SD = 3.09) on the depression scale (possible range 0–21), and 1.67 (SD = 1.85) on the anxiety scale (possible range 0–21), both of which indicate “normal” or very low levels of symptomatology. The mean score on perceived stress (PSS) was 13.11 (SD = 8.06), which, out of a possible range of 0–40, is considered “low stress”. The Rumination (RRS) mean score was 18.09 (SD = 5.07), out of a possible range of 10–40, indicating a low to moderate level of rumination. The suppression (ERQ) mean score was 13.88 (SD = 5.47) which, out of a possible range of 4–28, indicates a moderate level of suppression.

To test hypotheses one and two, bivariate correlation analyses were conducted between the ERQ (rumination), SF36 (health and well-being), DASS21 (depression and anxiety), RRS (rumination), and PSS (perceived stress) scores. Hypothesis one predicted that general health and well-being (SF36) would be negatively correlated with rumination (RRS); suppression (ERQ); and perceived stress (PSS). The results indicated that general health and well-being (SF36) were indeed negatively and moderately correlated with rumination (RRS), *r* (121) = −0.520, *p* < 0.05; and with perceived stress (PSS), *r* (121) = −0.673, *p* < 0.05; but was not significantly correlated with suppression (ERQ), *r* (121) = −0.081, *p* > 0.05.

Hypothesis two predicted that depression and anxiety scores (DASS21) would be positively correlated with rumination (RRS); suppression (ERQ); and perceived stress (PSS). The results indicated that depression (DASS21) was moderately and positively correlated with perceived stress (PSS), *r* (121) = 0.678, *p* < 0.05; and with rumination (RRS), *r* (121) = 0.547, *p* < 0.05; but weakly with suppression (ERQ), *r* (121) = 0.195, *p* < 0.05. Anxiety (DASS21) was weakly and positively correlated with rumination (RRS), *r* (121) = 0.483, *p* < 0.05; and with perceived stress (PSS), *r* (121) = 0.429, *p* < 0.05.

It is worth noting that the SF36 includes a 5-item subscale measuring Emotional Well-being, which overlaps with the DASS. This may have inflated the correlation between the general health and well-being scale with depression and anxiety (see Table 2 below).

A Multiple Regression analysis (MRA) was calculated to test hypothesis three, stating that general health and well-being (SF36) scores would be predicted by rumination (RRS); suppression (ERQ); and perceived stress (PSS). A significant regression equation was found (*F* (31, 17) = 35.459, *p* < 0.05), with an *R*^2^ of 0.476. It was found that rumination (RRS) significantly predicted general health and well-being (SF36) (β = −0.188, *p* < 0.05), as did perceived stress (PSS) (β = −0.563, *p* < 0.05), whereas suppression (ERQ) did not, (β = 0.005, *p* > 0.05). See Table 3 for more information.

Further MRAs were calculated to test hypothesis four, which stated that both depression and anxiety (DASS21) would individually be predicted by rumination (RRS); suppression (ERQ); and perceived stress (PSS). For depression, a significant regression equation was found (*F* (31, 17) = 39.698, *p* < 0.05), with an *R*^2^ of 0.504. A significant regression equation was found also for anxiety, (*F* (31, 17) = 14.932, *p* < 0.05), with an *R*^2^ of 0.277. Both rumination (RRS) and perceived stress (PSS) were significant predictors of depression and anxiety (DASS21), whereas suppression (ERQ) was not. See Table 3 for beta values and significance levels.

To further the analyses as a function of both physical and mental health, respondents were grouped into three distinct categories: those with a current diagnosis; those with a previous, but no current, diagnosis; and those with no diagnosis. To assess whether there were statistically significant differences between these groups, on the variables of interest (i.e., general health and well-being, rumination, suppression, perceived stress, depression, and anxiety) a series of Multivariate Analyses of Variance (MANOVA).

MANOVA—mental health diagnosis. The first MANOVA was conducted to assess whether there was a main effect of mental health diagnosis (current, previous, or none) on the dependent variables of interest. At the between-subjects level, all variables passed Levene’s Test for homogeneity of variance, apart from general health and well-being (SF36). As such, the variable of general health and well-being (SF36) was analysed separately using an independent samples *t*-test, which is discussed later in the results section.

A MANOVA was conducted. With the use of Pillai’s Trace criterion, the combined dependent variables were significantly affected by mental health diagnosis (current, previous, or none), *F* (12, 228) = 0.409, *p* = 0.000, partial η^2^ = 0.205.

Analysis of the dependent variables individually showed significant effect of mental health diagnosis for rumination, *F* (2, 118) = 7.139, *p* = 0.001, partial η^2^ = 0.108. The following were found via Tukey’s post hoc tests at the α = 0.05 level of significance: Participants with a current mental health diagnosis (M = 23.09, SD = 4.99) scored significantly higher on rumination compared to those with a previous diagnosis (M = 18.74, SD = 3.98) and to those with no history of mental health diagnosis (M = 17.35, SD = 4.96). Mental health diagnosis did not have a significant effect for suppression (*p* = 0.489), and the suppression means did not vary across mental health groupings in either direction.

MANOVA—physical health diagnosis. A second one-way between subjects MANOVA was conducted to assess whether there was a main effect of physical health diagnosis (current, previous, or none) on the dependent variables of interest. At the between-subjects level, all variables passed Levene’s Test for homogeneity of variance at α = 0.05, apart from rumination (RRS), *p* = 0.048.

A MANOVA was conducted. With the use of Pillai’s Trace criterion, the combined dependent variables were significantly affected by physical health diagnosis (current, previous, or none), *F* (12, 228) = 3.145, *p* = 0.000, partial η^2^ = 0.142.

Analysis of the dependent variables individually showed significant effect of physical health diagnosis for anxiety, (*F* (2, 118) = 6.846, *p* = 0.002) partial η^2^ = 0.104. Using Tukey’s post hoc tests at the α = 0.05 level of significance, it was found that participants with a current physical health diagnosis (M = 23.09, SD = 4.99) scored significantly higher on anxiety (M = 2.33, SD = 1.96) compared to those with no history of physical health diagnosis (M = 1.08, SD = 1.52). There was also a main effect of physical health diagnosis for general health and well-being, *F* (2, 118) = 7.09, *p* = 0.001, partial η^2^ = 0.107. Tukey’s post hoc tests found that participants with a current physical health diagnosis scored significantly lower on general health and well-being (M = 65.03, SD= 21.90) than those with no history of physical health diagnosis (M = 79.18, SD = 17.27).

Though non-significant results are not traditionally reported, the post hoc findings regarding rumination and suppression are worth mentioning, as these were the main variables under investigation for this study. In respect to rumination and suppression, physical health diagnosis did not have a significant effect on either variable. In fact, the mean score for rumination remained almost the same across groupings of physical health diagnosis (M = 18.45, SD = 0.73 for current diagnosis; M = 17.60, SD = 0.66 for no diagnosis). The same was found for suppression, with those reporting a current physical health diagnosis having a mean of 14.32 (SD = 0.78) on the suppression scale, and those with no diagnosis reporting a mean of 13.93 (SD = 0.71)

Independent samples *t*-test—mental health diagnosis. As Levene’s test showed that homogeneity of variance was violated for general health and well-being (SF36), the nonparametric alternative of Independent Samples Kruskal–Wallis test was conducted and the null hypothesis was rejected at α = 0.05. A series of three independent samples *t*-tests were conducted. Following, Bonferroni adjustment alpha was set at 0.017.

Equal variances were found for only one of the *t*-tests; this *t*-test compared participants with a current mental health diagnosis versus those with no history of diagnosis. Results from the independent samples *t*-test found that there was a statistically significant difference between scores on general health and well-being, *t* (100) = 7.05, *p* < 0.000 based on mental health diagnosis, with participants who had a current mental health diagnosis scoring significantly lower (M = 41.75, SD = 12.89) than those with no history of mental health diagnosis (M = 78.62, SD = 16.72).

## 6. Discussion

The purpose of the current study was to investigate the relationship between rumination, suppression, and perceived stress on physical and mental health and well-being in older adults. The results support the hypotheses in respect to the relationship between rumination and perceived stress with general health, mental health, and emotional well-being. However, the hypotheses regarding the relationship between suppression and general health, mental health, and emotional well-being were mostly refuted by the results.

### 6.1. Correlations with General Health and Well-Being

The results of the current study lend support to hypothesis one, which predicted a significant correlation between both rumination and perceived stress with measures of physical health and emotional well-being, as measured by the 36-item Health Survey subscales (SF36). However, there was no support for hypothesis two, which predicted that high scores on depression and anxiety would each be positively correlated with high scores on trait rumination, suppression, and perceived stress. Specifically, both rumination and perceived stress were significantly and negatively correlated with self-rated scores on general health and well-being, whereas suppression was not significantly correlated.

Rumination and perceived stress. The results showed a statistically significant relationship between high levels of habitual rumination and perceived stress, and lower levels of general health and emotional well-being in late adulthood. These findings are consistent with the rumination literature to date and seem to replicate the findings by [18] who conducted a similar study wherein older adults who habitually ruminated self-reported poorer physical health, and higher levels of arousal (stress).

Additional exploratory correlations were conducted, which found that rumination was moderately and positively correlated with perceived stress. Although the relationship between stress and general health and well-being has a long, established history [31,32], findings related to habitual rumination and general health and well-being outcomes in later life are far sparser. Also, the findings of a significant correlation between perceived stress and rumination are consistent with previous research findings that increases in rumination were predicted by higher levels of perceived stress, and equally, that rumination can generate and extend the stress response [21,22,23,24,37].

This correlation between rumination and perceived stress may help to explain a potential means through which rumination is negatively associated with later life health outcomes, i.e., via a heightened and prolonged experience of stress. To our knowledge, the current is only the second study to assess habitual rumination and stress and their relationship with physical health outcomes in older, non-clinical adults [18]. As such, the results of the current study extend our knowledge regarding the potential harmful correlates of habitual rumination on physical health and well-being in later life, and the parallel experience of heightened levels of stress.

Suppression, a non-significant correlation. The statistically non-significant relationship between suppression and general health and well-being found in the current study was surprising. Past research has suggested that suppression is related to lower reports of subjective well-being and may be associated with poor health outcomes, specifically a 10% increase in developing cardiovascular disease risk over a ten-year period [4], as well as significantly higher levels of inflammation than non-suppressors [38]. The results of the current study did not support these past research findings, however, this may be partly explained by the non-significant correlation between suppression and perceived stress, the rationale for which is explained next.

Past research has suggested that suppression may negatively impact health outcomes resultant of stress via intensified and prolonged physiological responding to stress and increased sympathetic activation of the cardiovascular system [1,6,7]. It is possible, then, that habitual suppression was not significantly correlated with self-rated health because participants did not experience concurrent heightened levels of stress. Indeed, the past research assessing the relationship between suppression and physiological stress responses have primarily explored the manipulation and/or induction of suppression, whereas the current study looked more broadly at habitual, naturally occurring suppression and more general health outcomes, rather than physiological measures of stress and poor health. This suggests that habitual suppression is not as deleterious on later life health outcomes as past research has suggested. This could be tested in future research whereby a mediation analysis with a larger sample could be conducted to help shed more light on the effects of suppression on health.

### 6.2. Correlations with Depression and Anxiety

Rumination and perceived stress. The results of the current study mainly support hypothesis two, which predicted significant correlations between both depression and anxiety scores with rumination, suppression, and perceived stress. Specifically, the results showed that depression was significantly, moderately, and positively correlated with perceived stress and rumination, and weakly, but significantly, associated with suppression. This means that high levels of depression were significantly correlated with higher scores on rumination, perceived stress, and to a lesser degree, with suppression. In respect to anxiety scores, the same trend was found, wherein higher scores on anxiety were significantly and positively correlated with higher scores on rumination and perceived stress. Anxiety, however, was not significantly correlated with suppression.

The above findings are consistent with past research, particularly in respect to the significant correlations between rumination and perceived stress with both anxiety and depression. These findings extend the previous literature that has primarily assessed these relationships in samples of college-aged students and adult clinical samples, by focusing on a community-based sample of older adults. Specifically, these results support the hypothesis that habitual rumination is associated with heightened levels of anxiety and depression in older adults.

Suppression. Interestingly, suppression was found to be significantly but weakly correlated with depression, whereas it was not significantly correlated with anxiety. These results are generally inconsistent with previous literature in respect to anxiety, which predominantly reported significant correlation between suppression and anxiety. However, the significant, though weak correlation between suppression and depression was consistent with previous findings [16].

In trying to understand the current results, it is important to consider that past research wherein significant correlations between suppression and psychopathology were found primarily studied this relationship in college-aged participants. The discrepancy in findings here suggests that suppression in older adults has less negative mental health outcomes than it does earlier in life, particularly in the form of anxiety. Considering that suppression was also found to have a non-significant correlation with perceived stress suggests that habitual suppression of emotional expression in later adulthood may not increase the subjective experience of stress. Perhaps it is via the subjective experience of stress that suppression is correlated with anxiety, and that the relationship between these variables becomes less significant when people are not experiencing stress. That is, perhaps, when people are not experiencing stress, the suppression of their emotional expression does not lead to heightened anxiety as there is conceivably less negative emotion to suppress, regardless of the habitual tendency to do so.

The weakly significant correlation between depression and suppression found in the current study suggests that a tendency to suppress emotions may not, in itself, contribute as significantly to depression when people do not perceive themselves to be under significant stress in the first place. Thus, the necessity to suppress the expression of their emotions is decreased, regardless of a habitual tendency to do so. In such case, participants may still self-rate themselves as high on habitual suppression, regardless of how frequently they have had to suppress strong emotions in the recent past. Perhaps it is the suppression of strong emotions that are associated with stress that contributes to depression and anxiety, whereas the habitual suppression of more common and non-distressing emotions is less maladaptive. Please note, however, that these are tentative hypotheses, which future research may test.

### 6.3. Predicting General Health and Well-Being

Hypothesis three was mainly supported, specifically in that general health and well-being scores were significantly predicted by scores on rumination and perceived stress, whereas suppression was not a significant predictor of general health and well-being. These findings extend the current literature in respect to rumination and its potential long-term effects on physical health and well-being. We are unaware of any studies to date (except for [18]) that have assessed a potentially predictive relationship between habitual rumination and self-rated physical health and well-being in older adulthood. It is important to note that these findings are based on multiple regression analyses with a cross-sectional design, therefore these results should be considered as preliminary. A longitudinal study regarding this predictive relationship would be valuable.

### 6.4. Predicting Depression and Anxiety

Hypothesis four predicted that depression and anxiety would each be significantly predicted by rumination, perceived stress, and suppression. Both rumination and perceived stress were indeed significant predictors of both anxiety and depression, whereas suppression failed to significantly predict either depression or anxiety. The non-significant findings regarding suppression and depression are particularly interesting as, suppression and depression were significantly, albeit weakly, correlated. In contrast, with rumination and perceived stress partialled out, the significant relationship between suppression and depression weakens to non-significance. This implies that the common variance between these variables is what is important in the association between suppression and depression. Although this was not further explored in the current study, it may be of interest in future research to expand with additional analyses.

As discussed previously, the association between rumination and perceived stress with anxiety and depression is consistent with previous studies. The added value from the current study is in relation to the sample’s characteristics, which comprised older, non-clinical sample of adult participants, rather than the more commonly surveyed college-aged and clinical samples primarily used in such research. Additionally, as a habitual rumination measure was utilized in the current study, the results suggest that both anxiety and depression are potentially precipitated by heightened levels of stress and a tendency to ruminate

### 6.5. Mental Health Diagnoses—Group Differences

Additional exploratory analyses were run, the results showed that respondents with current mental health diagnoses were significantly more likely to score higher on rumination than those with no history of mental health diagnoses. These results extend and validate the other findings of the current study that showed a significant correlation between rumination and higher self-ratings of anxiety and depression. Whether a participant has a current mental health diagnosis or not, is a more objective measure of current mental health than self-report as the respondent would have received their diagnosis from a health professional.

### 6.6. Limitations and Suggestions for Future Research

One of the major limitations is the correlational nature of the study, which limits the inferences that can be drawn from the findings. A longitudinal design may further test the hypotheses posed by the current study. This would allow researchers to ascertain whether participants maintained habitual engagement in rumination and/or suppression through their lifespan, and whether people who habitually ruminated/suppressed developed mental and physical health challenges in later years. This type of research was beyond the scope of the current study, hence, a cross-sectional survey design was utilised.

Another limitation is that, except for asking about medical diagnoses, the data are based on subjective reports, and did not include any objective physiological measures. However, as the interest of the current study was in exploring the relationship between trait rumination and trait suppression (i.e., not induced) with mental and physical health and well-being, a self-report survey design was appropriate.

The findings may also be limited in generalizability as most participants were in good health both physically and mentally. For example, 75.2% of participants reported having never had a mental health diagnosis, and average ratings on the DASS-21 for anxiety and depression were both low (in the ‘normal’, non-clinical range). In respect to physical health, the average self-ratings on general health and well-being across participants was 72.95 (SD = 20.51) of 100 (100 indicating ‘excellent health’), which suggests that the overall sample subjectively felt that they were in good health with very few physical limitations to their daily functioning. However, in comparing to US Norms for SF-36 within the age group of 65–74 years in 1993, the average score was 70.47, indicating that participants may in fact be quite representative of the general population. We did not find more recent SF36 norms internationally, nor Australian normative information for the SF36 based on age groupings.

In respect to the data, the ability to detect significant findings in the current study may have also been diluted because of the predominantly healthy sample of participants, and as such, there may be some additional significant relationships that were not detected in the current study. On average, participants also had very low to moderate scores on rumination, suppression, and perceived stress, which may also have limited our ability to detect significant relationships between these variables. The low average scores on anxiety, depression, and perceived stress may also decrease the generalizability of these results to populations with higher instances of psychopathology and stress. Obtaining such a healthy sample for the current study is, nevertheless, a strength as significant results were more difficult to obtain and can be considered valid as the hypotheses were put to a risky test.

The study would have been strengthened by mixed methods with follow up interviews or focus groups to determine the accuracy of self-reports. This was not feasible, however, as the study recruited an anonymous sample. Validation through follow up was not feasible for the same reason. This also meant that no clinical confirmation of self-report was possible.

Deviation from population data suggest that our sample was not representative of the population of older adult. Nevertheless, ours was an exploratory study offering preliminary findings toward larger scale research. Further, data related to ethnicity, educational level, income, employment status, history of PTSD, and/or immigration status, which would have strengthened the findings, were not collected. The use of MTurk for sampling may have introduced a sampling bias toward those who are tec savvy. Finally, data on physical activity, social isolation, or loneliness, were not gathered and were, thus, not used as covariates.

### 6.7. Implications

As the hypotheses regarding the maladaptive nature of rumination and perceived stress were generally supported, there are several suggested implications in respect to the potential long-term outcomes of habitually engaging in rumination as a way of regulating emotion. Mainly, these findings lend support to previous research that reported significant correlations between rumination, perceived stress, and physical as well as mental health and well-being. These results not only replicate and support past findings regarding the maladaptive nature of rumination but also extend the current knowledge by assessing these relationships within an older sample of community-based adults between the ages of 50 and 80 and focusing on habitual, rather than induced, rumination.

These findings suggest that continued research into the negative correlates of rumination would be advantageous. A more focused investigation of the potential causal relationship between rumination and poor physical and mental health would be particularly valuable. Being correlational, the current study limits causal conclusions that cannot make causal inferences based on its findings. Future longitudinal studies could investigate and clarify whether there is a causal relationship between rumination and mental/physical health outcomes in later life.

Findings from the current study suggest that people high in trait rumination may be at an increased risk of developing depression and anxiety symptoms, lower levels of subjective well-being, and a greater likelihood of developing physical symptoms in middle to late adulthood. These findings suggest that screening for trait rumination may be worthwhile in settings where people are facing mental or physical health challenges. Furthermore, the results indicate that continued development and implementation of targeted strategies and therapeutic techniques to reduce rumination may be worthwhile as both preventative and symptom-reduction approaches in the treatment of mental and physical health complaints. Some such approaches targeting ruminative thoughts and worry in the treatment of depression and generalized anxiety are already being developed and applied, particularly in the field of Cognitive Behavioural Therapy [37,39]. The results of the current study lend further support to this direction and focus of therapeutic intervention.

Of interest is the relative non-statistical significance of suppression in the current study. As mentioned earlier, the lack of significant findings is rather surprising as past literature has indicated significant relationships between suppression and an array of variables including depression, anxiety, well-being, markers for inflammation, and heightened and elongated stress responding. The current study failed to find any significant relationships between these variables and suppression, except for a weakly significant correlation between depression and suppression. It is suggested that further research, evaluating the relationship between these variables in older adults, would be helpful to elucidate whether these non-significant findings are replicable, and if so, whether there is an explanation for a potential shift in the maladaptive nature of suppression in late adulthood.

## 7. Conclusions

The conclusions that can be drawn from the findings of the current study are that rumination and perceived stress are not only significantly correlated with one another, but that each is significantly associated with self-rated general health and well-being, as well as depression and anxiety in later adulthood. The results also suggest that rumination and perceived stress may even predict heightened levels of anxiety and depression, and lower levels of general health and well-being. These findings suggest that trait rumination and stress may contribute to poor mental and physical health in later life. The results also suggest that the suppression of emotional expression is associated only with increases in depression, whereas suppression appears not to be strongly associated with anxiety, perceived stress, or general health and well-being in older adults.

Suggestions for future research include conducting longitudinal studies to further assess the associations between rumination, suppression, perceived stress, and mental and physical health and well-being. Additionally, future studies looking at trait rumination and trait suppression would benefit from including non-self-report measures, such as physiological or other objective measures. Considering the negative effects of prolonged stress on people’s immune system, the possibility that suppression, rumination, or self-perceived stress impact biological age would be of interest to future research.

The findings of the current study highlight the importance of conducting research with older adults in learning about both long-term effects of emotion regulation and the different effects such regulation mechanisms have across the lifespan.

## Figures and Tables

**Table 1:** Participant Physical and Mental Health Diagnoses.

Mental Health		Physical Health	
Diagnosis	Frequency	Diagnosis	Frequency
None	92	None	69
Depression	15	Hypertension	13
Multiple Diagnoses	7	Multiple Diagnoses	10
Anxiety	2	Arthritis	8
Bipolar Disorder	2	Cancers	5
PTSD	1	Diabetes	4
ADHD	1	Respiratory	3
OCD	1	Cardiac Conditions	3
		Neural Conditions	3
		Thyroid-related	1
		Colitis	1
		Osteoporosis	1

**Table 2:** Pearson Correlations.

Measure	1	2	3	4	5	6
1. Gen. Health	-	−0.520 **	−0.081	−0.673 **	−0.693 **	−0.507 **
2. Rumination	−0.520 **	-	0.087	0.592 **	0.547 **	0.483 **
3. Suppresion	−0.081	0.087	-	0.124	0.195 *	−0.051
4. Perceived Stress	−0.673 **	0.592 **	0.124	-	0.678 **	0.429 **
5. Depression	−0.693 **	0.547 **	0.195 *	0.678 **	-	0.395 **
6. Anxiety	−0.507 **	0.483 **	−0.051	−0.429 **	0.395 **	-

Note. Gen. Health = General Health and Wellbeing; ** = Correlation is significant at the 0.01 level (2-tailed); * = Correlation is significant at the 0.05 level (2-tailed).

**Table 3:** Multiple Regression Analyses: Beta values, significance levels, unstandardized coefficients, and standard errors.

	DV: Depression				DV: Anxiety				DV: Health & Well.			
IV	Beta	Sig.	B	Std. Err.	Beta	Sig.	B	Std. Err.	Beta	Sig.	B	Std. Err.
Perceived Stress	0.534	0.000	0.205	0.031	0.234	0.019	0.054	0.022	−0.563	0.000	−1.433	0.212
Rumination	0.221	0.007	0.135	0.049	0.354	0.000	0.129	0.036	−0.188	0.026	−0.759	0.336
Suppresion	0.109	0.098	0.248	0.149	−0.111	0.164	−0.150	0.107	0.005	0.938	0.079	1.012

Note. Sig. = Significance; DV = Dependent Variable; IV = Independent Variable; B = Unstandardized Coefficient; Std. Err. = Standard Error; Health & Well. = General Health & Wellbeing.

## Data Availability

Data may be obtained from the corresponding author by request.
